# Administration of perivascular cyanoacrylate for the prevention of cellular damage in saphenous vein grafts: an experimental model

**DOI:** 10.5830/CVJA-2015-078

**Published:** 2016

**Authors:** Nail Kahraman, Gunduz Yumun, Arif Gücü, Kadir Kağan Özsin, Taner Temmuz, Mehmet Tuğrul Göncü, Şener Ebru

**Affiliations:** Bursa Yuksek Ihtisas Education and Research Hospital, Bursa, Turkey; Namik Kemal University, Tekirdag, Turkey; Department of Cardiovascular Surgery, Bursa Yuksek Ihtisas Education and Research Hospital, Bursa, Turkey; Department of Cardiovascular Surgery, Bursa Yuksek Ihtisas Education and Research Hospital, Bursa, Turkey; Department of Cardiovascular Surgery, Bursa Yuksek Ihtisas Education and Research Hospital, Bursa, Turkey; Department of Cardiovascular Surgery, Bursa Yuksek Ihtisas Education and Research Hospital, Bursa, Turkey; Department of Pathology, Erzurum Education and Research Hospital, Erzurum, Turkey

**Keywords:** cyanoacrylate, saphenous vein graft, vascular damage, arterial pressure

## Abstract

**Objective:**

The saphenous vein is the most commonly used graft in coronary artery bypass surgery, since no suitable arterial graft is available. However, the frequency of late graft failure is a cause for research into graft protection. The objective of this study was to investigate the effect of synthetic adhesive cyanoacrylate administration on the saphenous vein graft for preventing vascular damage due to internal pressure on the graft.

**Methods:**

In this study we enrolled 20 volunteer subjects who had undergone coronary artery bypass surgery and who had excess saphenous vein grafts. Perivascular cyanoacrylate was administered to one of two saphenous vein grafts explanted from each patient. The other saphenous vein graft from each patient was not treated and was used as the control. A model of the arterial system was created using a saphenous vein cardiopulmonary bypass system. Circulation was maintained at 120 mmHg for 45 minutes. Afterwards, the grafts were subjected to histopathological examination.

**Results:**

The cyanoacrylate group of grafts did not develop severe vascular damage compared with many instances of moderate and severe damage due to compression in the control group of grafts (*p* = 0.003).

**Conclusion:**

Perivascular administration of cyanoacrylate appeared to be successful in the prevention of early saphenous vein graft injury. No *in vivo* study has been performed to date to assess endothelial damage in the saphenous vein, in order to demonstrate the long-term effect of cyanoacrylate. Further investigations are needed in this regard.

## Objective

Coronary artery bypass surgery (CABG) in coronary artery disease affords a longer lifespan for patients and higher patency rates of grafts than with percutaneous procedures.^1^,^2^ At present, the saphenous vein is the most commonly used graft, as no suitable arterial graft can be prepared for all vessels.

At the end of 10-year follow-up studies, it was reported that up to 40% of the saphenous vein grafts had undergone occlusion.^3^,^4^ Stenosis in up to half of the patent vessels is a significant negative factor with regard to long-term results after CABG. Therefore, a wide range of investigations has been conducted to investigate protection of saphenous vein grafts.

Increases in pulsatile flow and wall tension occur in venous grafts exposed to post-operative arterial pressure. Therefore injuries develop in the wall layers of the blood vessels, particularly the endothelium. Consequently, proliferation and migration of cells, vascular smooth muscle hyperplasia, and the formation of myofibroblasts, as well as neo-intimal development occur.^5^-^7^

Numerous studies have been performed involving the use of rigid and elastic supports or fibrin glue for the outside of the venous grafts in order to reduce the stress on them.^8^-^11^ The objective of this study was to investigate the protective effect of perivascular cyanoacrylate administration against saphenous vein damage due to blood pressure in patients after CABG.

## Methods

The study included 20 of the patients who underwent on-pump CABG operations between 2011 and 2012 in our clinic, who agreed to participate in the study, and who had sufficient saphenous vein grafts for use in this study. Two graft samples were taken from each patient to ensure standardisation between the groups. Then each patient’s two grafts were divided into two groups, one with and one without perivascular cyanoacrylate application, to ensure each group had 20 grafts.

Patients who had undergone off-pump surgery, those who had had an emergency operation, those with insufficient saphenous vein grafts, those who did not agree to participate, those with known malignancies, and those with haematological problems were excluded from the study.

**Fig. 1.**
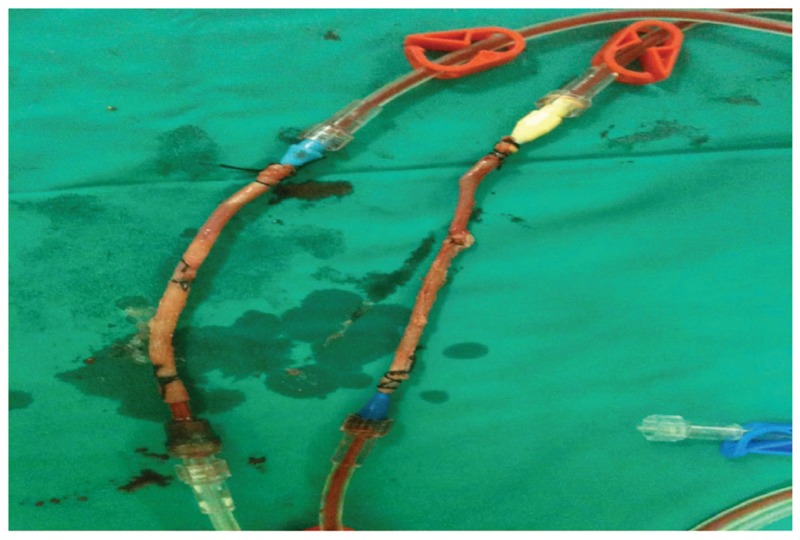
Perivascular cyanoacrylate applied to one of the saphenous vein grafts, prepared from the same patient, for the purpose of external support..

An arterial system model was created using redundant saphenous vein grafts in a cardiopulmonary bypass system (CPB). A device with two 3-cm-long saphenous vein grafts was created during CPB using a new line from the arterial line of the pump (Fig. 1). Cyanoacrylate was sprayed on the outer surface of one of the saphenous vein grafts and allowed to dry for five minutes. The other saphenous vein graft was not subjected to any process and both grafts were exposed to pump flow at 120 mmHg for approximately 45 minutes. At the end of the procedure, both saphenous veins were placed into containers with 10% formaldehyde solution and dispatched to the laboratory for histopathological examination.

## Histopathological examination

The removed grafts were fixed in 10% buffered formaldehyde solution for 24 hours. After routine tissue processing, 5-μm sections were cut from the paraffin-embedded blocks. These sections were stained with haematoxylin and eosin (H&E), histochemically with Masson’s Trichrom, and immunohistochemically with CD34, which is an endothelial marker. All sections were coded and the endothelium was examined under a light microscope by a pathologist who was unaware of the treatment protocol applied (Olympus CX 51, Tokyo, Japan).

**Fig. 2.**
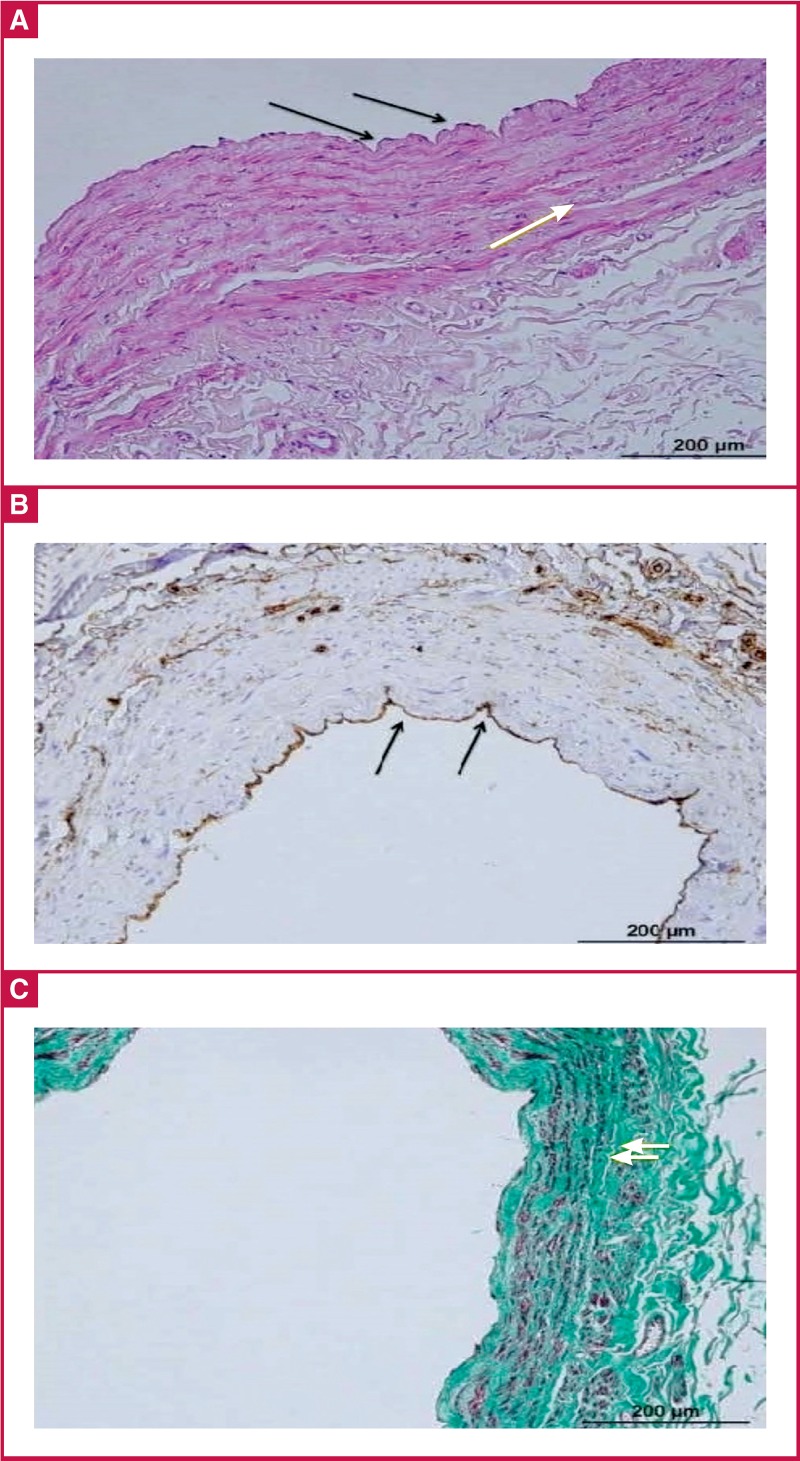
A shows mild endothelial cell loss (black arrows) with no oedema and no minimal intimal separation (white arrow). B shows CD34-labelled endothelial cell loss (black arrows). C shows minimal separation of the tunica media and intima but no loss of organelles.

**Fig. 3.**
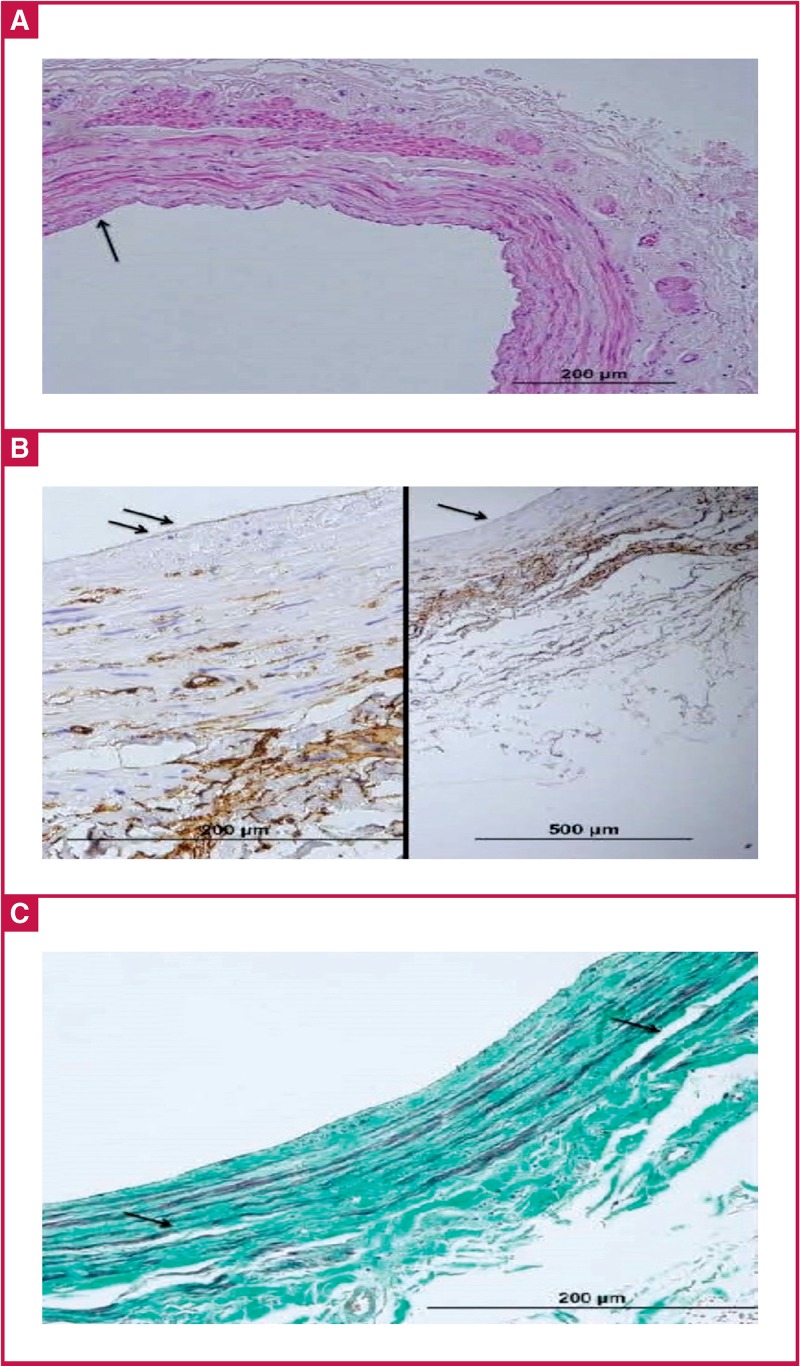
A shows moderate endothelial cell loss (black arrows). B shows moderate loss of CD34-labelled endothelial cells (black arrows). C shows significant separation between the tunica media and intima (black arrows), and oedema (white arrow). Additionally, there is mild loss in the organelle distribution in the collagen fibres.

**Fig. 4.**
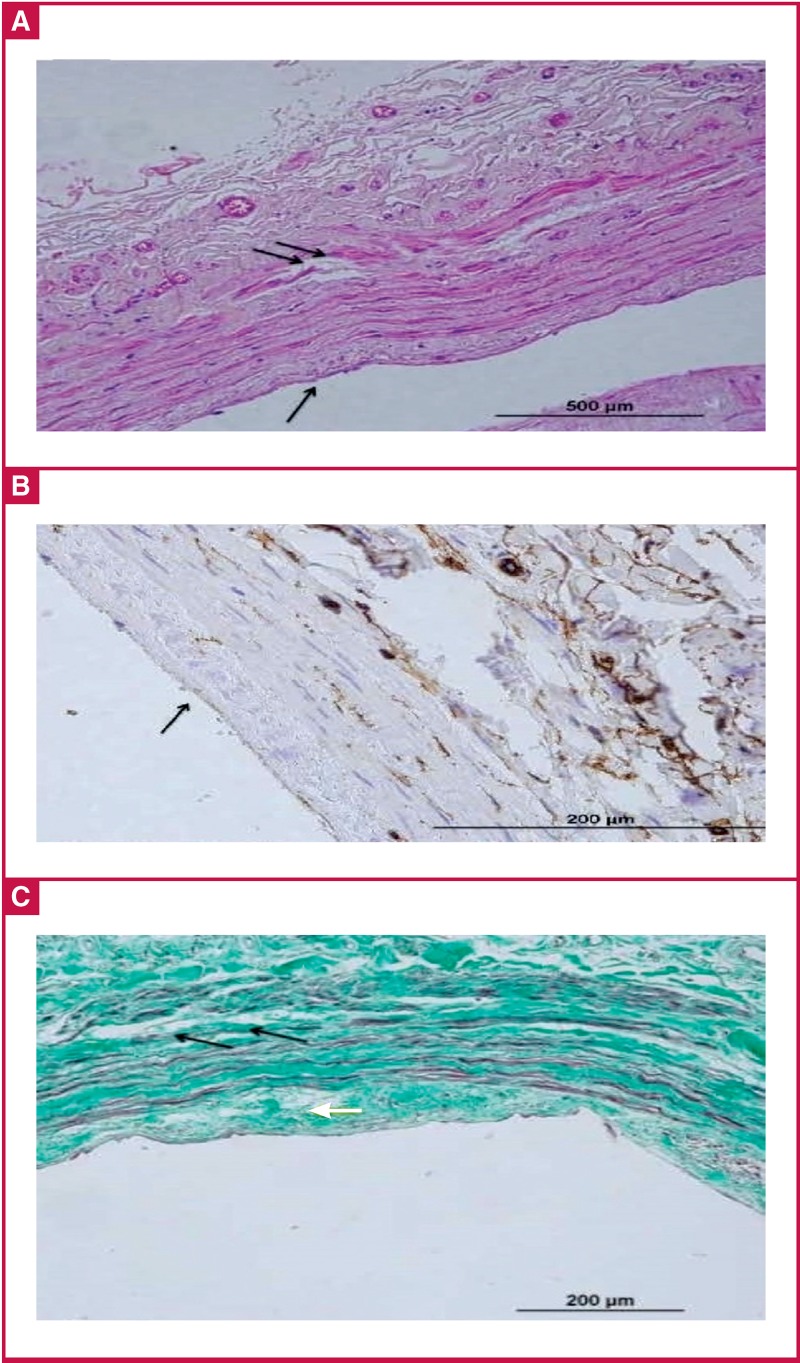
A shows severe endothelial cell loss (black arrows). B shows nearly total loss of CD34-labelled endothelial cells (black arrows). C shows significant separation between the tunica media and intima (black arrows), and oedema (white arrow). Additionally, there is loss in the organelle distribution in the collagen fibres..

The histomorphological classification of endothelial injury was as follows:

No injury: endothelial cells are in contact with each other, the cell has no change in contents or reduction in diameter. Platelets and other blood cells may or may not have adhesions to the endothelium (Fig. 2).
Type 1 injury: while the integrity of endothelial cells is maintained on the entire endothelial surface and endothelial cells are in contact with each other, there is a change in contents and reduction in diameter (flattening) of the cell. There is adhesion of the platelets and other blood cells to the endothelium (Fig. 2).
Type 2 injury: there is detachment of the cells from the junctions and lack of endothelial cells in places (Fig. 3).
Type 3 injury: there is endothelial cell peeling and the subsequent formation of sub-endothelial tissue (Fig. 4).

## Statistical analysis

The variables obtained were classified into categories and indicated as numbers and percentages. The chi-squared test was used to evaluate the analysis of categorical data. SPSS 18 (SPSS Inc, Chicago, IL, USA) software was used for statistical evaluations.

## Results

Endothelial injury was determined from the diameter of the saphenous vein, which remained unchanged in the group supported with cyanoacrylate, whereas severe distention of the saphenous vein occurred in the control group.

Endothelial injury was examined by H&E staining and immunohistochemically with CD34 staining, which is an endothelial marker. On first impression, no severe damage was seen in the saphenous vein grafts from the cyanoacrylate group, whereas severe endothelial injury and tunica media defects were seen in the control group.

Within the frame of classification of endothelial injury, in the control group, no significant injury was observed in three samples, whereas type 1, type 2 and type 3 vascular endothelial injury was seen in six, six and five grafts, respectively. In the cyanoacrylate group, no endothelial injury was observed in seven grafts, type 1 and type 2 endothelial injury was seen in 10 and two grafts, respectively, and there was no type 3 injury in the grafts. Endothelial injury was significantly less in the cyanoacrylate group, as shown by the assessment of intergroup results (Table 1).

**Table 1:** Distribution of saphenous vein injury in the groups as per classification

*Class of vascular damage*	*Group 1 (perivascular cyanoacrylate)*	*Group 2 (control)*	*p-value*
No injury	7	3	0.003
Type 1 injury	10	6	
Type 2 injury	2	6	
Type 3 injury	0	5	

The cyanoacrylate group did not exhibit any significant change in the medial layer of the saphenous vein grafts, whereas Masson’s Trichrom staining demonstrated significant separation and oedema between the tunica media and intima in the untreated saphenous vein graft group.

## Discussion

In this study, a model of the arterial system was established and the saphenous vein graft was exposed to internal pressure. Perivascular administration of cyanoacrylate onto the saphenous vein graft to prevent vascular injury due to extreme stress in group 1 proved to be protective *in vitro*.

CABG remains the superior method for promoting quality of life and lifespan in the treatment of coronary artery disease affecting multiple vessels. Long-term success is determined by graft success. Atherosclerosis and early occlusions may be seen in a short period of time, particularly in venous grafts.

Increased rates of atherosclerosis and rapid neo-intimal hyperplasia in venous grafts are described as endothelial injury and endothelial dysfunction as a result of exposure of the vein to the arterial system in peri-operative graft preparations.^3^-^5^ In recent years, pharmacological, genetic and physical protective methods have been described to provide protection against early injury to saphenous vein grafts.^3^-^15^

Ip *et al.* classified coronary endothelial injury into three types and reported that particularly type 3 injury may result in stenosis and occlusion of the coronary artery.^6^ Endothelial injury was defined as: type 1 injury: normal morphology despite functional changes in the endothelial layer; type 2 injury: maintenance of internal elastic lamina and medial layer despite separation, local peeling and intimal damage in the endothelial layer; type 3 damage: peeling of the endothelial layer and subsequent formation of sub-endothelial tissue, and intimal and medial damage in the respective classification.

Okazaki *et al.*^17^ classified endothelial injury into five stages. The classification includes stage 1: normal morphology; stages 2 and 3: minor or diffuse adhesion of blood cells (corresponding to type 1 injury); stage 4: rare isolated separation in endothelial cells (corresponding to type 2 injury); stage 5: generalised lack of endothelial cells (corresponding to type 3 injury). In particular, development of type 3 (stage 5) injury and widespread formation of a sub-endothelial layer will lead to platelet aggregation and the formation of thrombus as a result of contact between blood components and this layer. This will trigger smooth muscle proliferation and migration with mitogen factors; hence this may result in early or late stenosis or occlusion in the anastomosis area.^16^-^17^

Stooker *et al.* exposed saphenous veins to an arterial systemlike pressure atmosphere using a non-pulsatile cardiopulmonary device. The study indicated that endothelial injury of the saphenous vein graft was prevented by fibrin glue.^10^

Fibrin and fibrinogen degradation products proved to be potent chemotactic agents for saphenous vein grafts.^14^ However, in a report by Nomura *et al.* regarding the use of perivascular fibrin glue, they state it may direct the cellular stimulus and migration to the adventitial level, preventing intimal thickening.^15^ Perivascular fibrin glue and losartan administration were demonstrated by Moon *et al.* to prevent neo-intimal hyperplasia following saphenous vein graft angioplasty.^18^

Absorbable (vicryl) and non-absorbable (polyester) loose stents significantly reduced neo-intimal thickening, as shown by the examination of saphenous vein grafts at one and six months.^6^,^9^ Looseness of the support was reported as the potential reason.^7^ However, the space between the graft and these loose grafts was filled with fibrin after a short time and the loose structure disappeared, stimulating new microvascular growth in that area.^9^,^19^ It has therefore been suggested to cause development of the vasa vasorum and protection against vascular hypoxia, not intimal thickening.

However, in an *in vivo* study by Wan *et al.*, saphenous vein graft intimal thickening could not be reduced by fibrin glue in the long term.^5^ The reason is that the chemotactic effects of fibrin cannot be controlled and it will stimulate late intimal thickening.

We did not discover any other studies on this topic, apart from cyanoacrylate-related vascular embolism and arterial intervention-site repair.^20^,^21^ These resources specify the superior adhesion characteristic of cyanoacrylate and its low fibrosis rate during the follow-up period.

Dai *et al.* showed reduced intimal and medial thickening of the vein graft and inflammatory responses in their rabbit model study.^22^ However, it is not known whether the use of cyanoacrylate *in vivo* will cause graft restriction and related problems. This is because previously used supportive stents were loose or absorbable, whereas cyanoacrylate is a strong adhesive expected to have long-term durability.

## Conclusion

This study demonstrated that cyanoacrylate provided external support to the graft without any chemotactic effect, and primary protection of the graft against damage due to extreme stress. However, *in vivo* studies are required to investigate the effects on the graft in the long term.
